# The importance of observing the master’s hand: Action Observation Training promotes the acquisition of new musical skills

**DOI:** 10.3389/fneur.2024.1383053

**Published:** 2024-05-30

**Authors:** Simone Paolini, Maria Chiara Bazzini, Laura Ferrari, Antonino Errante, Leonardo Fogassi, Giacomo Rizzolatti, Maddalena Fabbri-Destro, Pietro Avanzini, Arturo Nuara

**Affiliations:** ^1^Dipartimento di Medicina e Chirurgia, Università di Parma, Parma, Italy; ^2^Consiglio Nazionale Delle Ricerche, Istituto di Neuroscienze, Parma, Italy; ^3^School of Advanced Studies, Center for Neuroscience, Università di Camerino, Camerino, Italy; ^4^Dipartimento Diagnostico, Unità di Neuroradiologia, Azienda Ospedaliero-Universitaria di Parma, Parma, Italy

**Keywords:** mirror mechanism, motor learning, motor similarity, musical training, music pedagogy

## Abstract

**Introduction:**

Via mirror mechanism, motor training approaches based on the alternation of action observation and execution (i.e., Action Observation Training-AOT) promote the acquisition of motor abilities. Previous studies showed that both visual and auditory stimuli may elicit a common motor representation of music-related gestures; however, the potentialities of AOT for the acquisition of musical skills are still underexplored.

**Methods:**

Twenty-one music-naïve participants underwent two blocks of training: AOT and Key-light Observation Training (KOT). AOT consisted of the observation of a melodic sequence played on a keyboard with the right hand by an expert model, followed by participant’s imitation. Observation and execution were repeated six consecutive times (T1–T6). KOT followed the same procedure, except for the visual content of the stimulus, depicting the sequential highlighting of the piano keys corresponding to the melody. The rate of correct notes (C), the trainee-model similarity of key-pressure strength (S), and the trainee-model consistency of note duration (R) were collected across T1–T6.

**Results:**

Both AOT and KOT improved musical performance. Noteworthy, AOT showed a higher learning magnitude relative to KOT in terms of C and S.

**Discussion:**

Action Observation Training promotes the acquisition of key elements of melodic sequences, encompassing not only the accurate sequencing of notes but also their expressive characteristics, such as key-pressure dynamics. The convergence of listening and observation of actions onto a shared motor representation not only explains several pedagogical approaches applied in all musical cultures worldwide, but also enhances the potential efficacy of current procedures for music training.

## 1 Introduction

The proficiency of musical performance largely depends on the fine concertation between the cortical and subcortical structures subserving the movement coordination in space and time (1). Some of these brain centers–especially those within frontal and parietal cortices–may also be active when no overt action is intended. This is the case of action observation (2), which is known to evoke the motor representation of the observed action in the perceiver’s motor system. Such motor resonance, termed *mirror mechanism* (3, 4), largely relies on the motor capacities of the observer, with actions belonging to the observer’s motor repertoire determining a stronger mirror response (5, 6). Interestingly, such dependency between the individual motor competencies and the mirror responsiveness seems reversed when moving from passive action observation to observation for later imitation. In this case, brain areas endowed with mirror properties are more activated for novel than familiar actions (7, 8).

Beyond its exquisite neurophysiological value, the mirror mechanism constitutes the theoretical ground prompting the development of a wide range of practices based on the systematic observation of actions followed by their immediate reproduction (Action Observation Training - AOT) (9, 10). In the design of the AOT interventions, motor imagery is often used as an intermediate stage connecting the initial action observation with the ultimate execution. This approach follows the organizational principle of AOT, i.e., the idea that observation provides an exogenous input to the motor system, and imagery makes subjects endogenously rehearse that program without the constraints of their peripheral capacities, with their chaining setting the ideal premises for better execution (10). AOT has proven effective in tuning existing motor competencies in fields requiring precise motor control, such as sports, and promoting the recovery of motor abilities in many clinical conditions (9–11). However, systematic and controlled evidence of the AOT efficacy in promoting musical learning is still lacking.

Despite this lack of knowledge, several traditional musical training methods vastly relied on the coupling between the observation of music gestures and their corresponding execution. Exemplar is the case of the first stages of instrumental training, where pupils are requested to observe and then imitate the master’s hand movements to shape a proper manual posture (12). Such an imitative scaffold is even more emphasized in ancient musical traditions such as Hindustani musical system, where music is mostly orally transmitted from masters to pupils (10, 13), as well as in training methods like Suzuki’s, in which the preliminary stage of learning is named “*minarai kikan*” [literally “period of learning by watching” (10, 13–15)].

Given these premises, we designed a behavioral study to demonstrate the efficacy of AOT in promoting the learning of a musical piece at the piano. In such a context, trainees are required to achieve three main outcomes: (1) correctly chain a determined sequence of keys (i.e., the proper note sequencing), (2) convey proper sound dynamics (from *piano* to *forte*), and (3) hold each note for a proper time duration, i.e., to get rhythm. Given such a multifaceted nature of musical execution, an open point remains on which components of the musical performance take more advantage of AOT.

To tackle these issues, 21 music-naïve participants underwent piano-playing training sessions based alternatively on AOT or the simple melodic reproduction instructed by visual cues. Melodies were performed on a MIDI musical keyboard, allowing the tracking of several parameters over time, including the sequence, strength, and timing of the pressed keys. We expected that observing the master’s hand would provide an add-on stimulation of the motor system via *mirror mechanism* and boost the musical learning outcome. If this is the case, our study will open to the incorporation of AOT in music pedagogy as an “extra weapon,” capable not only of ameliorating the appraisal of melodic sequences but also of transferring from the model (master) to the observer (pupil) the dynamics of the motor performance that holds the colors of music.

## 2 Methods

### 2.1 Participants

Twenty-one music-naive, right-handed [Edinburgh Handedness Inventory (16)] healthy participants (mean age 28 ± 6, 11 males) were enrolled. No subject had ever practiced music with any musical instrument, either at an amateur or professional level. The local ethics committee approved the study (Comitato Etico dell’Area Vasta Emilia Nord, n. 10,084, 12.03.2018), which was conducted according to the principles of the Declaration of Helsinki. The participants provided their written informed consent.

### 2.2 Experimental design

**Figure 1:**
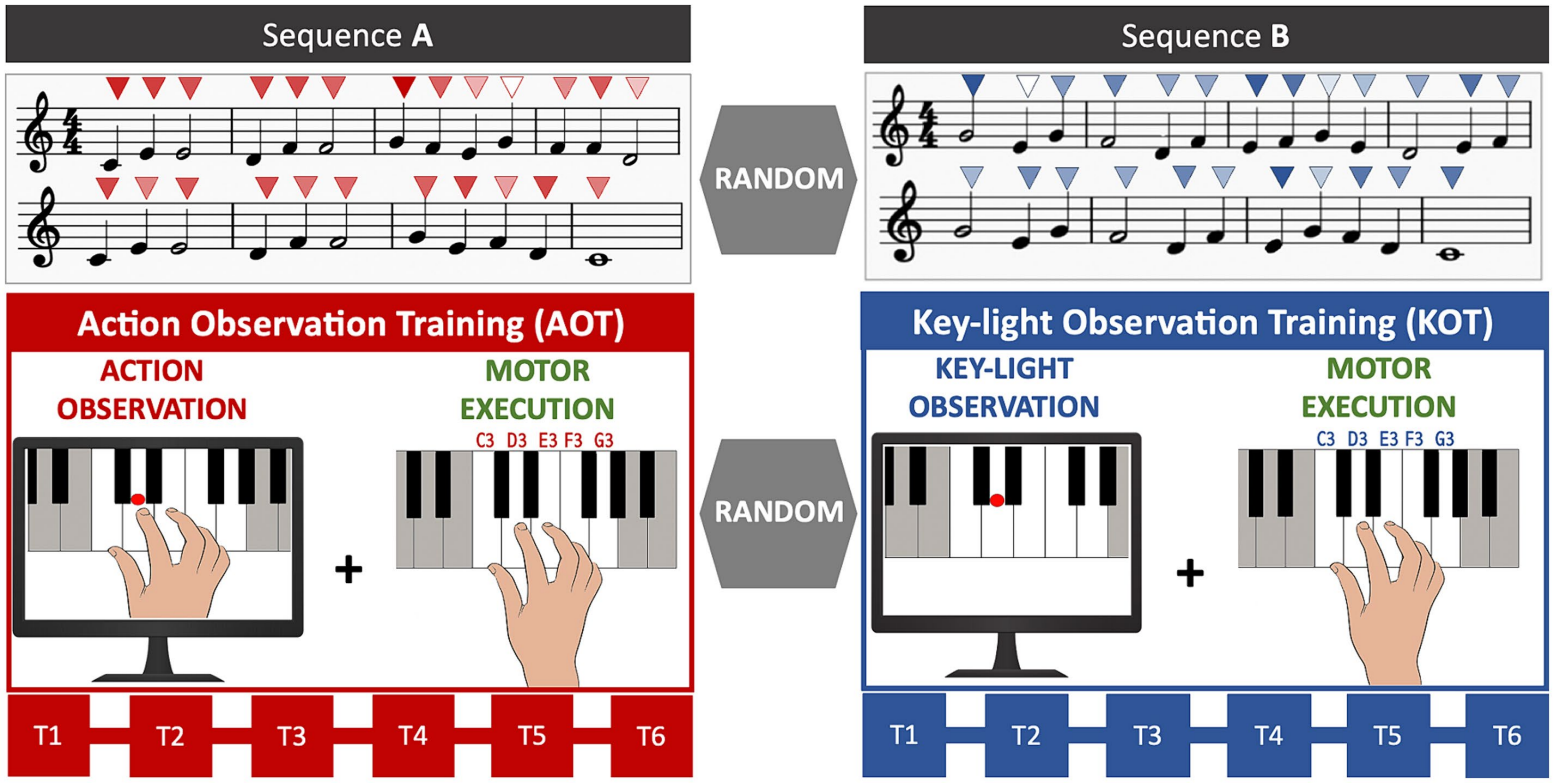
Experimental design. AOT consisted of the observation of a simple melodic sequence played on a keyboard with the right hand by the model, followed by its execution. Observation and execution were repeated six consecutive times (T1–T6). KOT followed the same procedure, except for the visual content of the stimulus, which depicted the sequential highlighting of the piano keys corresponding to the same melody used in AOT. Both training blocks (AOT vs. KOT) and sequences (A vs. B) were administered in randomized order across participants. Above each note, the colour intensity of the triangles (from light to dark) indicates the corresponding sound intensity (from *piano* to *forte*).

Each participant underwent two consecutive training blocks (randomized order): Action Observation Training (AOT) and Key-Light Observation Training (KOT), as depicted in Figure 1. AOT was based on the observation of a video clip depicting, from an egocentric perspective, a pianist playing with the right hand a piece of music (see Supplementary Videos 1, 2). The piano keys corresponding to the melody where highlighted during the video. The “first-person” perspective was chosen since it is more informative about the played keys, when compared with a frontal/lateral allocentric view, as well as for its acknowledged capacity to induce a higher reactivity of fronto-parietal circuits during action observation (17–19). Immediately following observation, participants were asked to execute the same melody. The KOT procedures were identical, except for: (1) the visual content of the stimulus, here depicting only the sequential highlighting of the piano keys corresponding to the melody, (2) the administration of a different melody to practice. Observation and execution were repeated six consecutive times (T1–T6) for each training. Within training sessions, real-time auditory feedback was delivered, including the specific tones corresponding to each key press (i.e., the pitch) and the dynamic range of the sound (i.e., the intensity of sound). The inclusion of auditory feedback consistent with the observed stimulus, is supported by previous studies evidencing that an ecological action-effect congruence may enhance the learning of new melodic sequences (20).

The melodic sequences for AOT and KOT were randomly selected from two melodies, balanced in terms of difficulty (number of notes = 24, rhythmic figures: 18 quarter notes, 6 half notes, 1 whole note; speed = 90 beats per minute). Notes, ranging from C3 to G3, were played according to the following constrained fingering: thumb-C3; 2nd finger-D3; 3rd finger-E3; 4th finger-F3; little finger-G3 (see Figure 1). Participants’ and model’s performance were acquired via a MIDI keyboard (Alesis V61) connected to a PC. Logic Pro App (Apple Inc., Cupertino, California) was used as recording software. This setup allowed us to digitally record the played notes as well as capture their timing and key-pressure strength.

### 2.3 Performance scoring

Participants’ performance was evaluated for each training block (T1–T6), considering the following domains:

#### 2.3.1 Note accuracy

The hit rate was calculated as the percentage (H%) of notes correctly executed by the trainee out of the total notes constituting the model melody (*n* = 24); the wrong note rate was computed as the percentage (W%) of incorrect notes out of the total notes played by the trainee. Finally, the relative amount of correct notes out of the total executed notes [C = H%/(H% + W%)] was chosen as the main outcome of note sequence accuracy, ranging from 0 (i.e., the subject executes only wrong notes) to 1 (i.e., all the performed notes are correct). In addition, the correctness of the sequence was assessed by means of a sliding window over 3 consecutive responses (“triplets”), following the procedure described in Sakreida et al. (21). Here, the successful execution of any three consecutive responses in the proper order, was classified as one accurate triplet. Then, the total number of accurate triplets was set as endpoint (C_T_).

#### 2.3.2 The key-pressure strength

The key-pressure strength (KPS) was transduced by the digital keyboard and sampled according to the 128-point MIDI protocol (22). To normalize the inter-subjects and inter-sequence force differences, the KPS of each correct note performed by each subject was expressed as its ratio with the maximum value within each melodic sequence (i.e., the most strongly played note having KPS = 1). The same procedure was then applied for characterizing the dynamic of KPS of the model. Finally, the trainee-model KPS absolute difference (*d*) was computed for the correct notes, the trainee-model similarity (*S*) of key-pressure strength was derived using the formula *S = 1−d,* and then expressed in percentage. An S index of 100% indicates an identical dynamic of KPS between the trainee and model, while lower percentages indicate a trainee dynamic increasingly detaching from model one.

#### 2.3.3 Note duration

The trainee-model absolute difference of note duration (ms) was computed for each correct note, and its mean value (within-session) was chosen as the rhythmic outcome (*R*).

An exemplar extraction of the variables above from a single performance (Subject 9, T5, melodic sequence A) is detailed in Supplementary Material 1.

### 2.4 Statistical analyses

The homogeneity of basal (T1) performance between training types (AOT vs. KOT) for the different behavioral variables (i.e., C, S, R) was assessed by paired-sample t-tests. Moreover, to exclude a different degree of difficulty between the two melodies (A and B), their basal performance was compared with a paired-sample *t*-test. Finally, to assess if there is an effect of the first training on the second, we compared the basal performance (C,S,R) of the second block of training between participants that underwent AOT or KOT first.

A mixed rmANOVA was applied to investigate the effect of time (T1–T6) and training type (AOT vs. KOT) on the performance outcomes (C, S, R). In case of significant main effects or interactions, post-hoc analysis was conducted, and a Bonferroni correction was applied to account for planned, pairwise comparisons. The same analyses above were applied to the secondary outcomes (H%, W%, C_T_), whose results are displayed in the Supplementary Material 2. The significance threshold was set at 5%.

## 3 Results

The basal performance did not differ either between AOT and KOT, and between melodic sequences, indicating a homogeneous starting level for the musical performance. The order of training did not affect the participant’s performance either for C or S, but for note duration R. In particular, subjects undergoing AOT-first, showed an increased R in the following, basal performance of KOT (596 ms *VS* 1055 ms; *t* (40) = −4.63, *p* < 0.001).

**Figure 2:**
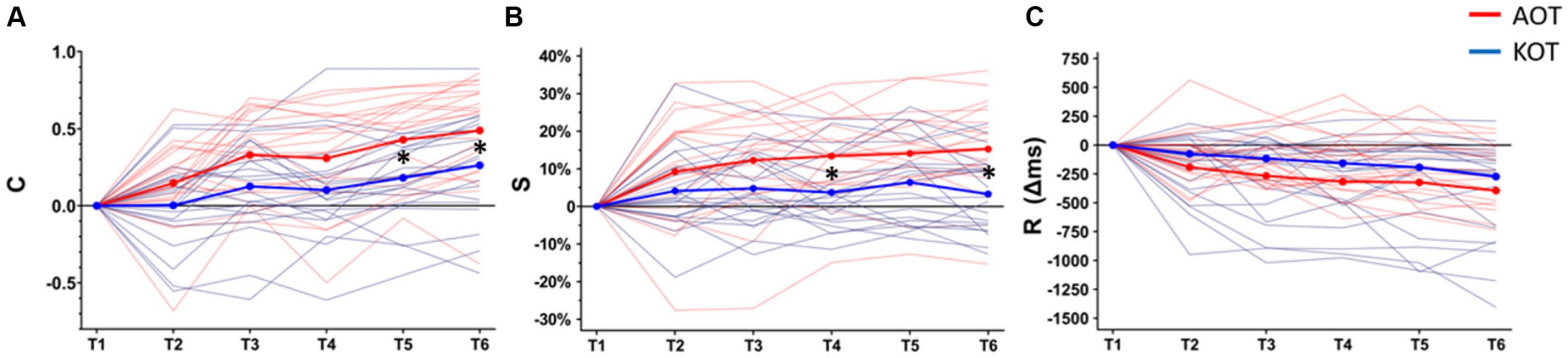
Changes across the evaluation timepoints (T1–T6) of the three main outcomes: Percentage of correct notes out of the total executed notes **(***C*, panel **A)**, Trainee-model key-pressure strength similarity **(***S*, panel **B)**, mean difference (Δ ms) of note duration between trainee and model **(***R*, panel **C)**. All means are baseline (T1) corrected. Single-subject learning trajectories and mean values are represented in thin and thick lines, respectively. Asterisks indicate the level of significance in between-groups comparisons across timepoints. AOT, Action Observation Training; KOT, Key-light Observation Training.

The values of all the performance indexes across timepoints are shown in Supplementary Table 1. Baseline-corrected values of the three main outcomes across timepoints (T1–T6) are depicted in Figure 2. The rmANOVA showed a significant main effect of TIME on C (*F* (5,100) = 11.67, *p* < 0.001, *η*_p_^2^ = 0.579) and no effect of TRAINING (*F* (1,20) = 2.96, *p* = 0.100, *η*_p_^2^ = 0.129). A significant TIME x TRAINING interaction emerged (*F* (5,100) = 3.29, *p* = 0.009, *η*_p_^2^ = 0.157), with post-hoc comparisons revealing that AOT training induced a significant advantage at T5 and T6 relative to the same time points for KOT (AOT vs. KOT, T5: 68% ± 24% vs. 54% ± 20%, *p* = 0.013 and T6: 74% ± 25% vs. 61% ± 22%, *p* = 0.034).

Concerning key-pressure strength (S), a similar pattern emerged from the rmANOVA, with a significant effect of TIME (*F* (5,100) = 15.26, *p* < 0.001, *η*_p_^2^ = 0.432), no effect of TRAINING (*F* (1,20) = 1.47, *p* = 0.238, *η*_p_^2^ = 0.068), and a significant TIME x TRAINING interaction (*F* (5,100) = 4.07, *p* = 0.002, *η*_p_^2^ = 0.169). Post-hoc comparisons indicated that AOT training outscored KOT at T4 and T6 (AOT vs. KOT, T4: 86% ± 5% vs. 81% ± 7%, *p* = 0.042; T6: 87% ± 4% vs. 81 ± 8%, *p* < 0.001).

Finally, for the note duration (*R*), the rmANOVA showed a significant effect of TIME (*F* (5,100) = 18.11, *p* < 0.001, *η*_p_^2^ = 0.475), but no significant effect of TRAINING (*F*_(1,20)_ = 0.22, *p* = 0.642, *η*_p_^2^ = 0.011) or TIME x TRAINING interaction were found (*F* (5,100) = 0.97, *p* = 0.438, *η*_p_^2^ = 0.046). In other words, both musical trainings affected the trainee-model deviation in note timing, and apparently, the improvement was larger for AOT relative to KOT. However, this difference did not reach the significance threshold.

## 4 Discussion

In recent years, a growing body of research has pointed out that motor training based on action observation boosts the improvement of motor skills in a wide range of human activities (10, 23, 24). In the present study, we investigated whether the same potentiality can be applied also to the music realm and, if this is the case, which aspects of musical performance were mostly impacted. Musical tasks employed in our study are different from simple motor sequences commonly employed in serial reaction time tasks. Indeed, their inherent musical properties, including rhythm, pitch, and dynamic variation, require the integration of sensory feedback with precise motor output to produce not just a sequence of actions but a coherent and expressive musical piece.

Concerning note accuracy, AOT, on average, enhanced participants’ learning by 30% relative to KOT, in line with previous experiments showing that–when coupled with action execution–action observation drives the acquisition of new procedural tasks [e.g., tying nautical knots (23) or learning abstract visuo-spatial sequences (25–27)]. However, most of the AOT studies published to date employed motor tasks providing participants with only visual cues. The learning paradigm employed in our study is different, because both AOT and KOT include the notion of the key sequence linked to acoustical feedback, yet with the former task adding the view of the *master’s hand*. The superiority of AOT in promoting musical learning suggests that, in addition to a possible effect elicited by the observation of the sequence of keys, another factor comes into play. More specifically, the observation of the action executed by the master triggers, in addition to the simple sequence of visual cues, a motor representation of congruent finger movements, polarizing the trainee’s motor system - and thus his motor attempt - toward the master’s performance. It is worth mentioning that when dealing with complex, sequential actions (like piano playing), action observation does not activate a single set of motor programs in the observer’s brain; instead, it engages the whole chain of individual motor acts composing that action. This motor resonance matching the temporal organization of the action has been demonstrated both in single-neuron studies in non-human primates (28) and in intracranial studies in humans (29), providing a neurophysiological substrate of why action observation could improve the motor learning outcome by prompting a motor program already possessing its inherent spatiotemporal complexity.

Motor resonance is not limited to what the master is doing but also to his kinematics (30–32). To quantify the adherence of the trainee to master’s dynamics, we measured the key-pressure strength similarity, revealing that AOT exerted on this domain its most selective advantage. Indeed, while control training did not induce any significant modulation over time, AOT was able to polarize the trainee’s expressive pattern toward those of the observed model. Such a “dragging effect” is consistent with recent experiments showing that the improvement of the motor performance induced by AOT is linked to the increase of similarity of the observer’s motor pattern toward the model (24). The observation of hand-keys interaction, coupled with prominent acoustical information, may have sculpted a more comprehensive motor representation, in turn promoting a more similar motor performance. This hypothesis is also supported by pivotal studies on observational learning showing that mere visual information can promote the acquisition of neural representations of novel force environments (33), as well as by previous evidence on AOT capability to modulate muscular strength during object manipulation (34–36). In this regard, the possibility of observing a biological effector rather than non-biological visual cues may have provided an additional advantage to the trainee in terms of imitative performance (37).

Concerning the accuracy in note duration, both training significantly improved this aspect over time, with AOT bringing a slight yet non-significant advantage. Although we cannot draw a firm conclusion on this point, a possible explanation is that the auditory cues common to both AOT and KOT played a predominant role in shaping the temporal tuning of the trainee’s performance, while visual information added only a mild “temporal binding” advantage (38).

Features like the note intensity and duration constitute the major vehicle through which music drives or induces its affective content. According to Truslit (39), the “expressive shaping” of music occurs during both its execution and perception, this latter being characterized by the perceiver’s “inward experience of movement” (39, 40). Such an inner motor representation would emerge from the embodiment of *dynamics* (i.e., the gradations of sound intensity changing the volume of perceived sound) and *agogics* (i.e., the temporal fluctuations of sound). Looking at our findings from this perspective, it could be speculated that the embodiment of the dynamo-agogic features of perceived music may have promoted the behavioral convergence of our participants toward the model’s performance, finally improving the AOT outcome.

Even if the collection of neuroimaging data goes beyond the scope of this study, some theoretical speculations on the neural substrates of our findings may be advanced on the basis of previous literature. Learning to play a piece of music on a keyboard involves the activity of the dorsal (dPMC) and ventral premotor cortex (vPMC) (41), as well as structures like cerebellum (42, 43). Noteworthy, the perception of musical actions modulates the activity of these brain structures (41, 44), suggesting their potential involvement in learning the correct chaining of the notes. Moreover – even if not strictly in the framework of musical performance – neuroimaging studies showed that the frontoparietal mirror circuit is involved in the learning of both sequencing and rhythmic tasks, with the former relying more strongly on posterior parietal regions and the latter recruiting additional networks for encoding rhythmical information (21).

It is worth noting that multiple cortical hubs encoding musical actions–i.e., (1) the dorsal premotor cortex, (2) the inferior parietal lobule and (3) the insular cortex – show an increased activation when trainees listen to a just practiced melody (41, 45), suggesting that, since the initial phases of learning, the perception of musical stimuli may have enabled our participants to “internally” play the perceived melodies, even without moving the hands (10).

Besides the musicological framework, another point of interest is the possible exploitation of music-based AOT in clinical contexts where both AOT (9–11) and musical interventions (46–49) – even if in isolation– have been already fruitfully administered. By coupling the administration of the musical stimuli with their rehearsal before the active movement phase, music-based AOT would sustain the interplay across auditory, visual, and motor domains, sustaining activity-dependent neuroplastic changes associated with functional recovery (46). At the same time, MIDI protocol would easily allow the manipulation of distinct musical features (e.g., speed, timbre, loudness), adapting the stimuli to the specific needs of the patient. Not least of all, putting music into AOT would take advantage of the intrinsic motivational features of musical experience.

In conclusion, we provided the first evidence that Action Observation Training drives the acquisition of key elements of musical performance, encompassing not only the accurate sequencing of notes (i.e., the *content* of the melody) but also their expressive characteristics (i.e., the *color* of the melody). The convergence of listening and observation of actions onto a shared motor representation, enhances the potential efficacy of current methods for music education, explaining several pedagogical approaches that are fruitfully applied in all musical cultures worldwide.

## Data availability statement

The raw data supporting the conclusions of this article will be made available by the authors, without undue reservation.

## Ethics statement

The studies involving humans were approved by Comitato Etico dell’Area Vasta Emilia Nord. The studies were conducted in accordance with the local legislation and institutional requirements. The participants provided their written informed consent to participate in this study.

## Author contributions

SP: Data curation, Formal analysis, Writing – original draft, Writing – review & editing. MB: Data curation, Writing – review & editing. LFe: Data curation, Writing – review & editing. AE: Writing – review & editing. LFo: Writing – review & editing. GR: Writing – review & editing. MF-D: Conceptualization, Methodology, Writing – review & editing. PA: Conceptualization, Formal analysis, Methodology, Writing – review & editing. AN: Conceptualization, Data curation, Formal analysis, Methodology, Supervision, Writing – original draft, Writing – review & editing.
