# Supplementary material for: The importance of observing the master’s hand: Action Observation Training promotes the acquisition of new musical skills
Source: Front Neurol. 2024 May 30;15:1383053. doi: 10.3389/fneur.2024.1383053 (PMC11169796; doi:10.3389/fneur.2024.1383053)
Supplement: Supplementary file 1 [file Table_1.DOCX]

**Supplementary material 1**

**Exemplar extraction of the behavioral endpoints from a single performance (Subject 9, T5, melodic sequence A).** The upper figure shows a “piano-roll” view of both model and participant’s performance. The bottom table displays the note-by-note comparison between the model’s and participant’s performances in term of key-sequence, key-pressure strength, and duration.

**
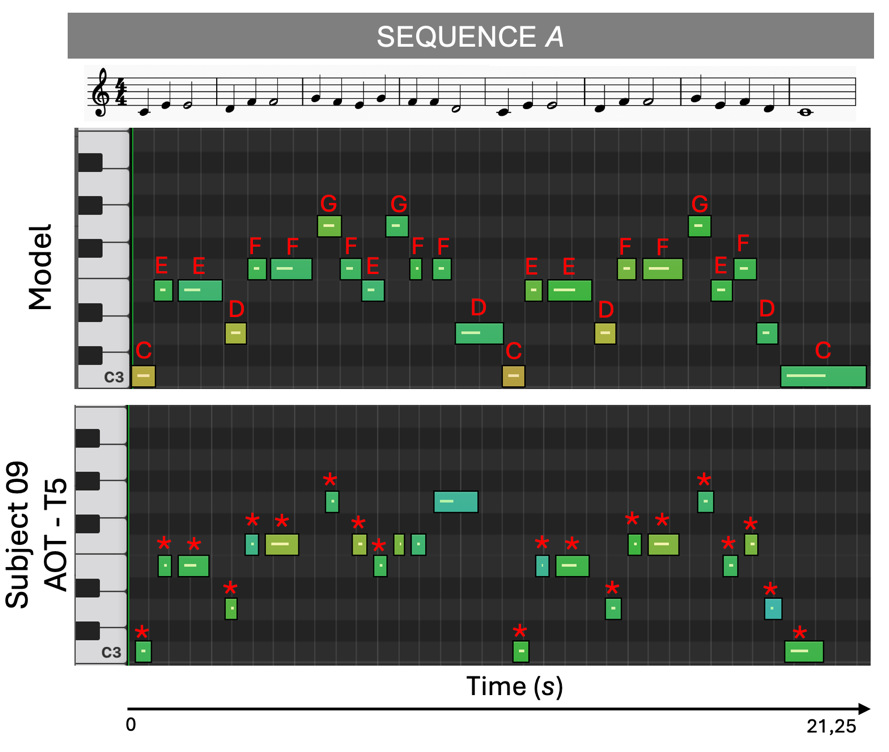

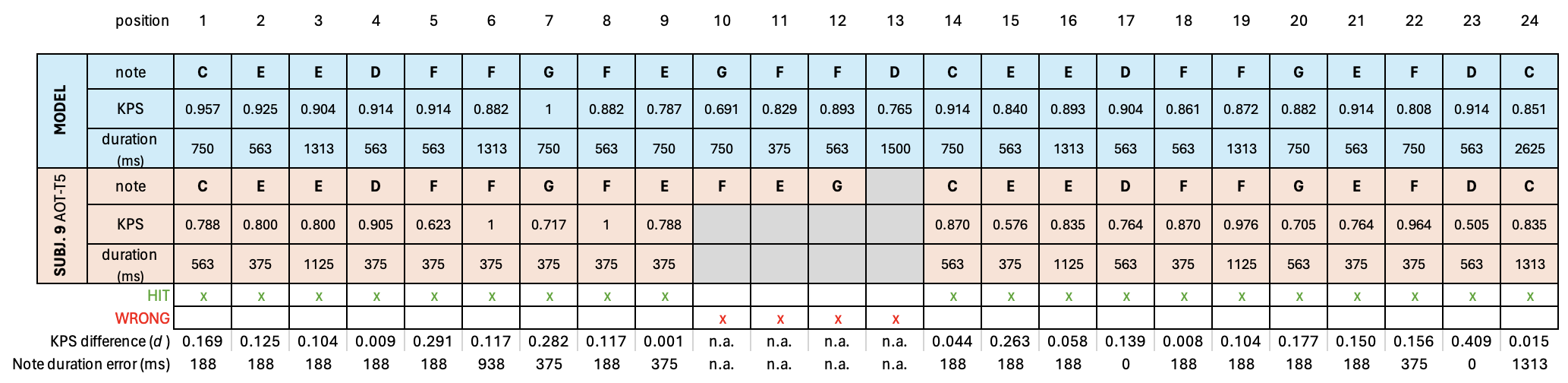
**

Mean Key Pressure Strength (KPS) difference (d) = 0.137

Mean Note duration error = 291 ms

Hit rate (H%) = 20/24 = 83.3%

Wrong notes (W%) = 3/23 = 13%

Consecutive correct triplets of notes (C_T_) = 16

**C**= H%/(H%+W%) = 86.5%

Trainee-model KPS similarity (**S**)= 1-d = 86.3%

The trainee-model difference of note duration (**R**) = 291 ms
