# Supplementary material for: The importance of observing the master’s hand: Action Observation Training promotes the acquisition of new musical skills
Source: Front Neurol. 2024 May 30;15:1383053. doi: 10.3389/fneur.2024.1383053 (PMC11169796; doi:10.3389/fneur.2024.1383053)
Supplement: Supplementary file 2 [file Table_2.docx]

**Supplementary material 2**

**Table 1.** Mean scores of behavioral indexes as H% (hit notes), W% (wrong notes), C (relative amount of correct notes), C_T_ (correct consecutive triplets of notes), S (trainee-model key-pressure strength similarity) and R (note duration error) across evaluation timepoints (T1-T6).

| **M ± SD** | | **T1** | **T2** | **T3** | **T4** | **T5** | **T6** |
| --- | --- | --- | --- | --- | --- | --- | --- |
| **H (%)** | ***AOT*** | 18.4±15.6 | 38.5±26.1 | 53.1±23.7 | 56.5±22.7 | 65.1±20.1 | 72.8±20.3 |
|  | ***KOT*** | 25.6±18.2 | 32.7±22.7 | 43.4±21.9 | 44±26.4 | 52.9±22.7 | 62.1±23.4 |
| **W (%)** | ***AOT*** | 50.2±51.3 | 54.1±58.2 | 47.4±72.9 | 52.5±65.7 | 44±63.2 | 41.2±73 |
|  | ***KOT*** | 43.6±50.2 | 57.5±69.9 | 51.7±60.6 | 64.5±57.8 | 59.9±64.6 | 48.6±53.8 |
| **C** | ***AOT*** | 36.6±26.5 | 46.3±22.8 | 61.1±23.8 | 57.1±24 | 68.1±24.6 | 74.1±25.5 |
|  | ***KOT*** | 47.9±27 | 43.3±23.4 | 52.5±23 | 43.2±25.6 | 54.6±21 | 61.7±22.6 |
| **C_T_** | ***AOT*** | 1.5±2.8 | 5.2±5.2 | 7.8±4.8 | 8.8±5.2 | 10.5±5.1 | 12.7±5.1 |
|  | ***KOT*** | 2.7±3.5 | 4.2±4.3 | 5.9±4.2 | 6.5±5.4 | 7.8±5.3 | 10.0±5.3 |
| **S (%)** | ***AOT*** | 72.7±10.8 | 81.9±8.1 | 84.9±7.3 | 86.1±5 | 86.8±3.7 | 87.9±3.8 |
|  | ***KOT*** | 78.1±12.6 | 82.2±9.8 | 82.9±8.3 | 81.8±7.6 | 84.5±4.8 | 81.4±7.8 |
| **R (Δms)** | ***AOT*** | 865.3±468.8 | 674.3±394.3 | 598.7±285.6 | 548.4±258.9 | 541.7±221.4 | 470.8±189.7 |
|  | ***KOT*** | 787.3±306.2 | 711±309.7 | 671.6±261 | 630.3±254.6 | 594.3±181 | 514.5±164.3 |

**Results of secondary outcomes measures**

A mixed rmANOVA was applied to investigate the effect of time (T1-T6) and training type (AOT vs. KOT) on the secondary outcomes (H%, W%, C_T_). In case of significant main effects or interactions, post-hoc analysis was conducted, and a Bonferroni correction was applied to account for planned, pairwise comparisons. Results are showed for the separate variables.

1. **H%**. The rmANOVA showed a significant main effect of TIME on performance (F(5,100)=65.99, p<0.001, ηp2=0.77) and no effect of TRAINING (F(1,20)= 2.32, p=0.14, ηp2=0.10). A significant TIME x TRAINING interaction emerged (F(5,100)=4.37, p=0.001, ηp2=0.18). Post-hoc comparisons showed a significant advantage of AOT vs KPT at T5 (65.1±20.1 vs 52.9±22.7, p=0.06).
2. **W%.** The rmANOVA showed a significant main effect of TIME on performance (F(5,100)=11.70, p<0.001, ηp2=0.37) and no effect of TRAINING (F(1,20)= 2.33, p=0.14, ηp2=0.10). Also, a significant TIME x TRAINING interaction was observed (F(5,100)=3.34, p=0.008, ηp2=0.14), but post-hoc comparisons did not show any significant results.
3. **C_T._** The rmANOVA showed a significant main effect of TIME on C (F(5,100)=53.63, p<0.001, ηp2=0.728) and no effect of TRAINING (F(1,20)= 2.05, p=0.166, ηp2=0.093). A significant TIME x TRAINING interaction emerged (F(5,100)=3.86, p=0.003, ηp2=0.162). Post hoc showed that AOT outperformed KOT in terms of correct, consecutive “triplets” (C_T_, T5: 10.5±5.1 vs 7.8±5.3, p=0.04 and T6: 12.7±5.1 vs. 10.0±5.3%, p=0.05).
